# Editorial: Lung Cancer—From Mechanisms of Action and Risk Factors in Disease Onset to Management

**DOI:** 10.3390/cancers18050874

**Published:** 2026-03-09

**Authors:** Irene Giacchetta, Roberto Fabiani

**Affiliations:** 1 Hospital Management of Maggiore and Bellaria, AUSL Bologna, Largo Nigrisoli, 40133 Bologna, Italy; 2 Department of Chemistry, Biology and Biotechnology, University of Perugia, Via del Giochetto, 06126 Perugia, Italy

## Abstract

Lung cancer is the leading cause of cancer-related mortality worldwide. This editorial accompanies the Special Issue “Lung Cancer: From Mechanisms of Action and Risk Factors in Disease Onset to Management” published in Cancers (MDPI), and introduces the twenty-one research and review articles included in the collection. The contributions span a wide spectrum of topics, from risk factors such as allostatic load and telomere biology, to molecular biomarkers including DNA methylation and serum glycopeptides, to advances in low-dose CT screening and the management of incidental findings, to targeted therapy, immunotherapy, surgical techniques, and health economics. Together, the papers highlight the multifactorial and clinically complex nature of lung cancer, and reinforce the importance of integrated, evidence-based strategies to reduce its global burden.

## 1. Introduction

Despite notable advances in prevention, early detection, and treatment over the past two decades, lung cancer remains the leading cause of cancer-related mortality worldwide, responsible for approximately 1.8 million deaths annually [1]. The disease encompasses a heterogeneous group of histological subtypes with distinct biological profiles, clinical behaviors, and therapeutic vulnerabilities. Its etiology reflects the interplay of behavioral exposures, environmental and occupational determinants, genomic susceptibility, and health system capacity. Addressing this burden requires coordinated effort spanning primary prevention, risk stratification, early diagnosis, precision therapy, and equitable access to innovation. The present Special Issue, published in Cancers (MDPI), brings together twenty-one peer-reviewed contributions that collectively span the full clinical and research continuum of lung cancer—from mechanisms of carcinogenesis to real-world management strategies. This editorial provides a thematic overview of the published works and contextualizes their contribution to the field.

## 2. Risk Factors, Allostatic Burden, and Biological Susceptibility

A central aim of this Special Issue was to advance understanding of the modifiable and biological determinants of lung cancer risk. Guan et al. examined the relationship between allostatic load—a composite measure of cumulative physiological stress—cigarette smoking, and lung cancer incidence [2]. Their findings suggest that chronic stress-mediated dysregulation of neuroendocrine and immune pathways may potentiate the carcinogenic effects of tobacco, reinforcing the importance of multidimensional risk profiling that extends beyond conventional exposure indices.

From a genomic stability perspective, Fabiani et al. conducted a systematic review and meta-analysis of prospective studies examining leukocyte telomere length (LTL) and lung cancer risk [3]. The analysis demonstrated a statistically significant inverse association between longer LTL and lung cancer incidence, consistent with the hypothesis that telomere attrition—reflecting accelerated cellular aging and impaired genomic maintenance—constitutes an independent risk modifier in lung carcinogenesis Together, these two contributions highlight the utility of integrating systemic biological markers alongside behavioral exposures in risk stratification models.

## 3. Molecular Biomarkers: Epigenomics, Proteomics, and Tumor Genetics

Several articles in this collection addressed the identification and validation of molecular biomarkers for lung cancer risk, early detection, and prognosis. Dolcini et al. performed a comprehensive systematic review and meta-analysis of DNA methylation biomarkers in lung cancer risk, analyzing data from multiple case–control and cohort studies [4]. Their results support a significant association between hypermethylation of selected tumor suppressor gene promoters in peripheral blood and elevated lung cancer risk, suggesting that circulating epigenomic signatures may serve as accessible, minimally invasive biomarkers for population-level risk screening.

In the domain of liquid biopsy and proteomics, Yamazaki et al. developed a novel approach combining comprehensive serum glycopeptide spectral profiling with machine learning algorithms for the early detection of lung cancer in a case–control setting [5]. Their model achieved promising discriminatory performance, indicating that glycoproteomic signatures may complement or enhance existing biomarker panels. Ko et al. further expanded the prognostic biomarker landscape by evaluating the role of CA-125 and CA-199—traditionally associated with gynecological and gastrointestinal cancers, respectively—in predicting outcomes in lung adenocarcinoma [6]. Their analysis demonstrated independent prognostic value for both markers beyond the established CEA threshold.

At the level of tumor molecular characterization, Bushara et al. described the clinical and molecular profile of SETD2-mutated lung adenocarcinoma, a genomic subtype defined by loss-of-function alterations in the histone methyltransferase gene SETD2 [7]. Their data revealed distinctive co-mutation patterns, immunological features, and clinical outcomes compared to SETD2 wild-type tumors, contributing to the growing map of actionable and prognostically relevant genomic alterations in NSCLC.

## 4. Lung Cancer Screening: Policy, Incidental Findings, and Histopathological Assessment

Screening represents one of the most impactful strategies for reducing lung cancer mortality through early-stage diagnosis. Firmani et al. provided a timely and comprehensive state-of-the-art review of lung cancer screening programs globally, mapping current policy frameworks, eligibility criteria, implementation challenges, and emerging evidence through 2025 [8]. Their work synthesizes data from major international trials—including the NLST and NELSON studies—and evaluates the translational gap between trial efficacy and real-world program effectiveness, with particular attention to disparities in access and adherence.

A complementary contribution by Lin et al. examined the clinical and management implications of incidental findings identified during lung cancer screening CT examinations [9]. Their analysis characterized the spectrum of non-lung-cancer findings—including cardiovascular, pulmonary, and extrathoracic abnormalities—and discussed evidence-based frameworks for their follow-up, highlighting the need for standardized protocols to optimize the benefit-to-harm ratio of screening programs. A subsequent correction to this work was also published within the Special Issue [9].

At the histopathological level, Lee et al. investigated the clinical significance of tumor spread through air spaces (STAS) grading in early-stage lung adenocarcinoma [10]. Their findings demonstrated that higher-grade STAS was independently associated with increased recurrence risk and poorer survival outcomes, supporting the integration of STAS assessment into routine pathological staging and surgical planning.

## 5. Precision Oncology: Next-Generation Sequencing and DNA Repair Targeting

The molecular characterization of lung tumors through next-generation sequencing (NGS) has become an essential component of treatment decision-making in NSCLC. Gaal et al. reported the genomic landscape of a Romanian cohort of NSCLC patients characterized by routine NGS testing using the Oncomine Dx Target Panel at a dedicated molecular pathology laboratory [11]. Their study identified the prevalence of clinically actionable alterations—including EGFR mutations, ALK and ROS1 fusions, KRAS mutations, and MET exon 14 skipping—in a population with limited prior genomic data, underscoring the importance of real-world NGS implementation in non-Western European settings and the need for equitable access to molecular diagnostics.

From a mechanistic and translational standpoint, Manguinhas et al. explored the potential of novel DNA repair inhibitors targeting XPG—a structure-specific endonuclease involved in the nucleotide excision repair pathway—to enhance cisplatin sensitivity in NSCLC [12]. Using combined in silico docking analyses and cell-based assays, their study identified candidate XPG-targeting compounds capable of potentiating platinum-induced cytotoxicity, providing a rational basis for further preclinical and clinical investigation of this therapeutic strategy.

## 6. Immunotherapy: Evidence, Real-World Data, and Biomarker Prediction

Immune checkpoint inhibition has transformed the systemic management of advanced NSCLC over the past decade. Three contributions in this Special Issue addressed distinct dimensions of immunotherapy from mechanistic, clinical, and translational perspectives. Roussot et al. provided a comprehensive review of immune checkpoint inhibitor strategies beyond the canonical PD-1/PD-L1 axis, examining emerging targets including CTLA-4, LAG-3, TIM-3, TIGIT, and their rational combination regimens [13]. The review contextualizes the biological rationale for dual and multi-checkpoint blockade and critically appraises current evidence from clinical trials, addressing mechanisms of primary and acquired resistance.

Marković et al. contributed real-world evidence on the efficacy of first-line pembrolizumab monotherapy in a multicenter cohort of metastatic NSCLC patients aged 70 years or older with high PD-L1 expression (TPS ≥ 50%) [14]. Their analysis, conducted across multiple European centers, demonstrated that clinical benefit was maintained in this elderly population, supporting the use of pembrolizumab as a standard first-line option regardless of age, while also identifying prognostic factors relevant to treatment selection in older patients.

Kokkotou et al. investigated the prognostic value of soluble PD-L1 and serum vascular endothelial growth factor-B (VEGF-B) in patients with advanced NSCLC receiving pembrolizumab [15]. Their data suggest that these circulating factors may independently predict clinical outcomes, offering potentially accessible and minimally invasive tools for treatment monitoring and prognostic stratification in immunotherapy-treated populations.

## 7. Brain Metastases: Real-World Outcomes in Resource-Limited Settings

Randjelovic et al. analyzed outcomes in a cohort of NSCLC patients with brain metastases treated at a center in a resource-limited country, providing a valuable real-world perspective that complements data from high-income settings [16]. Their study characterized patterns of care, treatment modalities employed, and survival outcomes, highlighting the persistent challenges of implementing evidence-based neurocognitive and systemic therapies in settings with constrained infrastructure and limited access to advanced treatment options. These findings reinforce the importance of international equity considerations in global lung cancer management guidelines.

## 8. Surgical Management: Resection Strategies, Carcinoids, and Postoperative Complications

Surgery remains the cornerstone of curative intent treatment for resectable lung cancer. Four surgical contributions in this Special Issue addressed the comparative efficacy of resection strategies, perioperative predictors of quality of life and overall survival, the long-term outcomes of lung carcinoids, and the management of a potentially life-threatening postoperative complication. Chiang et al. compared sublobar resection with lobectomy for small (≤3 cm) NSCLC presenting with visceral pleural invasion, using propensity-score matching on a large nationwide Taiwanese cohort [17]. Their analysis found that lobectomy was associated with superior overall and disease-free survival, even in this small-tumor subgroup with pleural involvement, providing important data to inform surgical decision-making in a histological scenario where the optimal extent of resection remains debated.

Complementing the analysis of resection extent, Fukai et al. conducted a prospective study examining pre- and postoperative predictors of health-related quality of life (HRQoL) and overall survival in patients undergoing surgery for NSCLC [18]. Their findings identified a set of socioclinical factors independently associated with perioperative HRQoL deterioration and long-term survival, underscoring that surgical outcomes in lung cancer extend beyond purely oncological endpoints. The study reinforces the importance of integrating patient-centered measures into preoperative assessment and shared decision-making frameworks, particularly in light of the growing recognition that HRQoL represents an independent and clinically meaningful determinant of postoperative recovery and prognosis.

Askildsen et al. examined recurrence rates and patterns following radical resection of lung carcinoids in a Scandinavian cohort [19]. Their long-term follow-up data demonstrated that while typical carcinoids carry a favorable prognosis after complete resection, atypical carcinoids showed substantially higher rates of late recurrence—including distant metastases—underscoring the need for extended postoperative surveillance protocols for this histological subtype.

Mazzella et al. provided a thorough clinical review of bronchopleural fistula (BPF) following lobectomy for lung cancer, one of the most severe and difficult-to-manage postoperative complications in thoracic surgery [20]. The authors systematically described the epidemiology, risk factors, diagnostic criteria, and a spectrum of therapeutic options ranging from conservative bronchoscopic techniques to innovative surgical and endoscopic interventions, providing a practical framework for multidisciplinary management of this life-threatening condition.

## 9. Health Economics: Comorbidity Patterns and Healthcare Costs

Buja et al. contributed a population-based economic analysis characterizing healthcare costs according to comorbidity patterns in lung cancer patients within the Veneto Region (Italy) [21]. Using administrative health data, the study identified distinct comorbidity clusters associated with markedly different healthcare resource utilization and expenditure profiles. These findings demonstrate that comorbidity-adjusted cost modeling is essential for accurate health economic evaluations of lung cancer care, and have implications for resource allocation, hospital reimbursement frameworks, and the design of integrated care pathways for patients with complex multimorbidity.

## 10. Conclusions

The twenty-one contributions assembled in this Special Issue collectively illustrate the breadth and depth of current lung cancer research, spanning biological mechanisms, molecular diagnostics, population-level screening, precision therapy, and health systems analysis. Several thematic threads emerge across the collection. First, the biological underpinnings of lung carcinogenesis extend well beyond tobacco exposure to encompass allostatic burden, epigenomic instability, telomeric aging, and tumor-specific genomic alterations. Second, the field is progressively moving toward minimally invasive biomarker strategies—circulating methylation signatures, glycoproteomic profiling, soluble immune checkpoints—that may enable earlier and more precise risk stratification and treatment monitoring. Third, real-world data are increasingly indispensable to complement randomized trial evidence, particularly for populations underrepresented in clinical trials, including elderly patients, those with significant comorbidities, and individuals in resource-limited settings.

Fourth, surgical decision-making continues to be refined through large retrospective and propensity-matched analyses that inform the optimal extent of resection across histological subtypes and anatomical scenarios. Alongside these oncological metrics, prospective evidence on perioperative quality of life and survival predictors is increasingly shaping a patient-centered approach to surgical care in lung cancer. Finally, health economics analyses remind us that clinical efficacy must be evaluated within the broader context of healthcare sustainability and equitable access.

We are grateful to all authors who contributed their work to this collection, to the reviewers whose critical expertise strengthened the scientific quality of each manuscript, and to the editorial team of Cancers (MDPI) for their support throughout the process. We hope that this Special Issue serves as a valuable resource for clinicians, researchers, epidemiologists, and health policy makers engaged in the global effort to reduce the burden of lung cancer.

## Data Availability

No new data were created or analyzed in this study. Data sharing is not applicable to this article.
